# Glomerular Filtration Rate and/or Ratio of Urine Albumin to Creatinine as Markers for Diabetic Retinopathy: A Ten-Year Follow-Up Study

**DOI:** 10.1155/2018/5637130

**Published:** 2018-02-26

**Authors:** Pedro Romero-Aroca, Marc Baget-Bernaldiz, Raul Navarro-Gil, Antonio Moreno-Ribas, Aida Valls-Mateu, Ramon Sagarra-Alamo, Joan F. Barrot-De La Puente, Xavier Mundet-Tuduri

**Affiliations:** ^1^Ophthalmology Service, University Hospital Sant Joan, Institut de Investigacio Sanitaria Pere Virgili (IISPV), Universitat Rovira & Virgili, Reus, Spain; ^2^Department of Computer Engineering and Mathematics, Universitat Rovira & Virgili, Reus, Spain; ^3^Health Care Area Reus-Priorat, Institut Catala de la Salut (ICS), Institut de Investigació Sanitària Pere Virgili (IISPV), Universitat Rovira & Virgili, Reus, Spain; ^4^Health Care Area Jordi Nadal de Salt (ICS), Unitat de Suport a la Recerca Barcelona Ciutat, Institut Universitari d'Investigacio en Atencio Primaria Jordi Gol (IDIAP Jordi Gol), Barcelona, Spain; ^5^Unitat de Suport a la Recerca Barcelona Ciutat, Institut Universitari d'Investigacio en Atencio Primaria Jordi Gol (IDIAP Jordi Gol), Universitat Autonoma de Barcelona, Bellaterra, Spain

## Abstract

**Aims:**

To determine the relationship between diabetic nephropathy and diabetic retinopathy on a population of type 2 diabetes mellitus patients.

**Methods:**

A prospective ten-year follow-up population-based study. We determined differences between estimated glomerular filtration rate (eGFR) using the chronic kidney disease epidemiology collaboration equation and urine albumin to creatinine ratio.

**Results:**

Annual incidence of any-DR was 8.21 ± 0.60% (7.06%–8.92%), sight-threatening diabetic retinopathy (STDR) was 2.65 ± 0.14% (2.48%–2.88%), and diabetic macular edema (DME) was 2.21 ± 0.18% (2%–2.49%). Renal study results were as follows: UACR > 30 mg/g had an annual incidence of 7.02 ± 0.05% (6.97%–7.09%), eGFR < 60 ml/min/1.73 m^2^ incidence was 5.89 ± 0.12% (5.70%–6.13%). Cox's proportional regression analysis of DR incidence shows that renal function studied by eGFR < 60 ml/min/1.73 m^2^ was less significant (*p* = 0.04, HR 1.223, 1.098–1.201) than UACR ≥ 300 mg/g (*p* < 0.001, HR 1.485, 1.103–1.548). The study of STDR shows that eGFR < 60 ml/min/1.73 m^2^ was significant (*p* = 0.02, HR 1.890, 1.267–2.820), UACR ≥ 300 mg/g (*p* < 0.001, HR 2.448, 1.595–3.757), and DME shows that eGFR < 60 ml/min/1.73 m^2^ was significant (*p* = 0.02, HR 1.920, 1.287–2.864) and UACR ≥ 300 mg/g (*p* < 0.001, HR 2.432, 1.584–3.732).

**Conclusions:**

The UACR has a better association with diabetic retinopathy than the eGFR, although both are important risk factors for diabetic retinopathy.

## 1. Introduction

Diabetic nephropathy (DN) and retinopathy (DR) are microvascular complications of diabetes mellitus which share risk factors, such as poor glycemia control and systolic hypertension. Their relationship has been the focus of our previous studies, where we demonstrate good positive relationship of microalbuminuria and macroalbuminuria in type 1 DM [1–3], despite in type 2 DM only macroalbuminuria was a significant risk factor in DR development [3].

The presence of diabetic retinopathy (DR) is easy to establish through observable retinal lesions. However, diabetic renal lesion is more difficult to diagnose. Nondiabetic nephropathy has a prevalence in DM patients that varies from different studies [4, 5] and difficult diagnosis of diabetic nephropathy. Diabetic retinopathy is present in T1DM with diabetic nephropathy [6]; on the contrary, type 2DM patients can develop DN without diabetic retinopathy [7].

Also, current clinical guidelines differ, such as KDIGO (Kidney Diseases: Improving Global Outcomes), who defines chronic renal diseases as the presence of an estimated glomerular filtration rate inferior to 60 ml/min/1.73 m^2^ or renal lesion that can be demonstrated from histological biopsy or by the ratio of urine albumin to creatinine (UACR) [8].

The recent introduction of estimated glomerular filtration rate (eGFR) has become an easy method for studying renal function, determined from serum creatinine, age, sex, and race [9]. At present, we have two major formulae, the equation of chronic kidney disease-epidemiology collaboration (CKD-EPI) [10] and the modification of diet in renal disease (MDRD-4 or MDRD-IDMS) [11]. Both can help us to determine renal function by a simple blood sample measure. The CKD-EPI equation is more accurate, especially with glomerular filtrate between 60 and 90 ml/min/1.73 m^2^ [12].

As we have previously published studies on DR incidence and its risk factors using data collected since 2007 from our screening programme of 15,811 Caucasian, type 2 DM patients [13], in the present study, we have the aim to determine whether there is a relationship between diabetic nephropathy and incidence of diabetic retinopathy in type 2 DM patients.

## 2. Materials and Methods

### 2.1. Setting

The reference population in our health care area (HCA) is 247,174 inhabitants. The current number of DM patients registered in our HCA is 18,528.

### 2.2. Design and Sample

This is a prospective, population-based study of 15,811 Caucasian, T2DM patients from data collected between 1st January 2007 and 31st December 2016, with a mean follow-up of 3.45 ± 1.12 times for each patient over the ten years. This includes 85.33% of T2DM patients of our HCA; the rest of the patients did not attend the screening, or they were lost during the study. Patients have been screened in our nonmydriatic fundus camera units (NMCU).

### 2.3. Power of the Study

Our epidemiologist evaluated the T2DM sample and estimated the accuracy of 95% with a ±3% increase in risk.

### 2.4. Method

Screening for DR was carried out with one 45° field retinograph, centred on the fovea. If DR was evident, another 2 retinographs of 45° were taken according to EURODIAB guidelines. The complete method is described elsewhere [14, 15].

In the present study, DR is thus classified as (i) no-DR = no diabetic retinopathy, (ii) any-DR = level 20 to 35 of the ETDRS, and (iii) STDR = level 43 or worse as defined by the ETDRS. The term diabetic macular edema (DME) includes extrafoveal and/or clinically significant macular edema (CSMO) according to the ETDRS classification [16].

Measures of renal diabetes disease were determined by

- Serum creatinine, determined by molecular absorption spectrometry,
- Estimated glomerular filtration rate (eGFR), calculated from plasma creatinine using chronic kidney disease epidemiology collaboration equation (CKD-EPI equation):
  1. For women
    - creatinine < 0.7 mg/dl eGFR = 144 × (creatinine/0.7)^−0.329^ × (0.993)^age^
    - creatinine > 0.7 mg/dl eGFR = 144 × (creatinine/0.7)^−1.209^ × (0.993)^age^
  2. For men
    - creatinine > 0.9 mg/dl eGFR = 141 × (creatinine/0.9)^−1.209^ × (0.993)^age^
- Gradation was expressed in ml/min/1.73 m^2^ and defined as normal = eGFR > 90, mildly diminished = eGFR 60–89, moderately diminished = eGFR 45–59, moderate–severely diminished = eGFR 30–44, and severely diminished = eGFR < 30.
- Ratio of urine albumin to creatinine (UACR). UACR was collected from urine samples for measurement of albumin and creatinine. Albumin was measured in mg/l and creatinine in mmol/l. The concentration ratio of urine to creatinine expressed in mg/g was used to estimate the total daily albumin excretion.
- UACR was classified as normoalbuminuria = UACR < 30 mg/g, microalbuminuria as UACR 30–299 mg/g, and macroalbuminuria as UACR ≥ 300 mg/g.
- The diagnosis of diabetic nephropathy was made by family physicians or endocrinologists, and according to the diagnosis, we use two different definitions of diabetic renal diseases:
  - Chronic kidney disease is defined as UACR ≥ 30 mg/g or eGFR < 60 ml/min/1.73 m^2^.
  - Renal failure is defined as UACR ≥ 300 mg/g and eGFR < 30 ml/min/1.73 m^2^.
- At the end of the study in January 2017, all electronic databases of patients were screened again to confirm the number of patients with DR and if any new patients had not previously been diagnosed.

### 2.5. Inclusion Criteria

Patients with all T2DM were screened in our primary HCA.

### 2.6. Exclusion Criteria

Patients with other specific types of diabetes and patients with gestational DM were excluded.

We followed our methods published by Romero-Aroca et al. [17].

### 2.7. Ethical Adherence

The study was carried out with the approval of the local ethics committee (approval number 13-01-31/proj6) and in accordance with revised guidelines of the Declaration of Helsinki. The study was approved and supported by Instituto de Salud Carlos III (IISCIII), Spain (FI12/01535, June 2013, and FI15/01150, July 15), and FEDER fundus.

### 2.8. Statistical Methods

The epidemiological risk factors included were as follows: current age, age at DM diagnosis, gender, duration and treatment of DM, arterial hypertension, levels of glycosylated haemoglobin (HbA_1c_), triglycerides, cholesterol fractions, and mentioned renal disease measures.

Incidence was measured for each year of the study using the following formula: *patients with DR/all patients screened.*

The ten-year sum incidence was calculated as *number of patients with DR over 10* years*/subjects at risk.*

Data evaluation and analysis was carried out using SPSS 22.0 statistical software package, and *p* < 0.05 was considered to indicate statistical significance.

Descriptive statistical analysis of quantitative data was made by determining mean, standard deviation, minimum and maximum values, and the 95% confidence interval. For qualitative data, we used the analysis of frequency and percentage in each category. Differences were examined using the two-tailed Student *t*-tests to compare two variables or using one-way ANOVA analysis of variance if we were comparing more than two variables. Inferential analysis for qualitative data was made by chi-squared table and by the Fisher test for quantitative data.

Survival analysis was carried out using the following:

- The Kaplan-Meier curve measured the accumulated risk of developing DR (in each of three forms: any-DR, STDR, and DME) in patients with renal failure using UACR ≥ 300 mg/g and using eGFR < 30 ml/min/1.73 m^2^.
- And the Cox proportional hazards model was used in order to evaluate survival curves considering other variables that may affect survival as age, sex, HbA1c, arterial hypertension, and DM treatment.

In Cox's survival analysis, we also applied four models:

1. Without renal status inclusion; no eGFR nor UACR included
2. Including eGFR < 60 ml/min/1.73 m^2^ as variable
3. Including UACR ≥ 300 mg/g as variable
4. Including eGFR < 60 ml/min/1.73 m^2^ and UACR ≥ 300 mg/g as variable

## 3. Results

### 3.1. Demographic Variables of Sample Size

In the ten-year follow-up (1 January 2007 to 31 December 2016), a total of 15,811 T2DM patients were screened (Table 1), which represents 85.33% of the total T2DM patients registered (18,528) in our health care areas (HCAs), with a mean follow-up of 3.45 ± 1.12 times for each patient over the ten years.

The whole sample included more males (56.13%), which does in fact reflect the prevalence of diabetes in the population as a whole. The mean current age was 63.91 ± 11.85 years and DM duration was 8.36 ± 6.64 years. Mean HbA1c values were 7.41 ± 1.45% (3.9–15.8). Excluding differences in age, men are more frequently being significant at *p* < 0.001.

Mean age of patients with any-DR was 63.91 ± 11.85 years, patients with STDR were 64.05 ± 12.27 years, and patients with DME were 64.36 ± 12.84 years.

### 3.2. Study of Incidence of Diabetic Retinopathy

A total of 4466 patients with T2DM developed any-DR (28.24%), with a mean annual incidence of 8.21 ± 0.60% (7.06%–8.92%) (Table 2). STDR in T2DM was developed in 1451 patients (9.17%) with an annual incidence of 2.65 ± 0.14% (2.48%–2.88%). DME in T2DM was developed in 1214 patients (7.67%) with an annual incidence of 2.21 ± 0.18% (2%–2.49%).

### 3.3. Study of Incidence of Nephropathy

A total of 3767 patients (23.82%) with T2DM had UACR > 30 mg/g with a mean annual incidence of 7.02 ± 0.05% (6.97%–7.09%) (Table 2). A total of 3173 patients had eGFR < 60 ml/min/1.73 m^2^ (20.06%) with an annual incidence of 5.89 ± 0.12% (5.70%–6.13%). Finally, 36 patients (0.22%) had renal failure, defined as UACR ≥ 300 mg/g and eGFR < 30 ml/min/1.73 m^2^ in T2DM, with an annual incidence of 0.06 ± 0.01% (0.04%–0.08%).

### 3.4. Statistical Analysis of any Diabetic Retinopathy

At the end of the study, all T2DM patients visited our unit and a fundus examination was carried out. We did not find any new patients with DR. We confirm, therefore, that no patient had been misdiagnosed during the screening programme. In the univariate analysis (Table 3), all studied variables are significant.

When we applied Cox's proportional regression analysis (Table 4), the variables studied in univariate analysis changed significance; thus, in the first model, current age, insulin treatment, arterial hypertension, and HbA1c levels sex were significant. The introduction of renal function causes new changes. The eGFR was significant in the second model (*p* = 0.002, HR 1.854), but when we introduced UACR in the third model, it became less significant (*p* = 0.05, HR 1.123). Finally, the inclusion of renal failure in the fourth model, defined as UACR ≥ 300 mg/g and eGFR < 30 ml/min/1.73, was significant at the same level as the UACR value.

### 3.5. Statistical Analysis of Severe Diabetic Retinopathy Forms

Table 5 shows descriptive data of different risk factors and its significance. In STDR, current age, DM duration, HbA1c levels, UACR, and eGFR are significant in two-tailed Student's *t*-test or using one-way ANOVA analysis of variance, but creatinine levels are not significant. Similarly, in the DME study, all previously described risk factors are significant in its development.

Table 6 shows significance of different variables in Cox's analysis. In STDR patients, all risk factors are significant in model 1, according to the hazard ratio HbA1c, UACR and arterial hypertension are important risk factors. In model 2 of STDR study, we substituted eGFR < 60 ml/min/1.73 m^2^ and UACR ≥ 30 mg/g for renal failure, defined as UACR ≥ 300 mg/g and eGFR < 30 ml/min/1.73 m^2^, which become an important risk factor with a hazard ratio of 3.174, like the hazard ratio of 3.230 of HbA1c.

The study of diabetic macular edema also demonstrated that all risk factors are significant in model 1, and HbA1c level, arterial hypertension, and UACR have the highest hazard ratio values. Also, the substitution of eGFR < 60 ml/min/1.73 m^2^ and UACR > 30 mg/g for renal failure becomes an important risk factor for DME development with a hazard ratio of 3.190 like 3.194 hazard ratio of HbA1c.

### 3.6. Study of Survival Analysis

**Figure 1:**
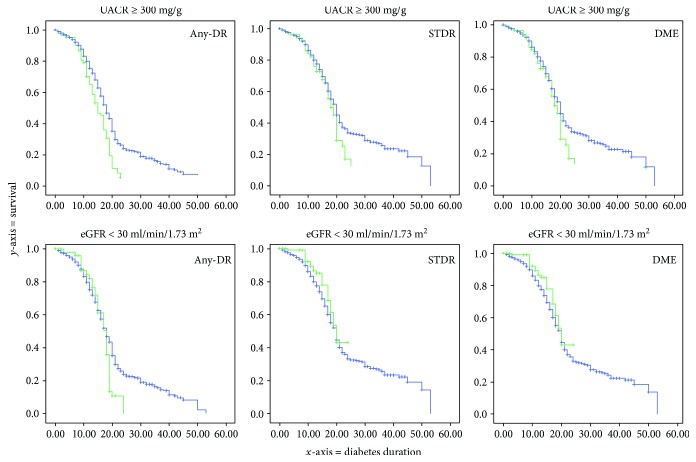
Plots of risk function of Kaplan-Meier study. The *y*-axis represents the accumulative risk to develop diabetic retinopathy, and the *x*-axis represents diabetes mellitus duration in years. The green curve represents effect of patients with renal failure as risk factor, and the blue curve patients without renal failure. At the top, there is the analysis of renal failure measured with UACR ≥ 300 mg/g, and at bottom eGFR < 30 ml/min/1.73 m^2^.

**Figure 2:**
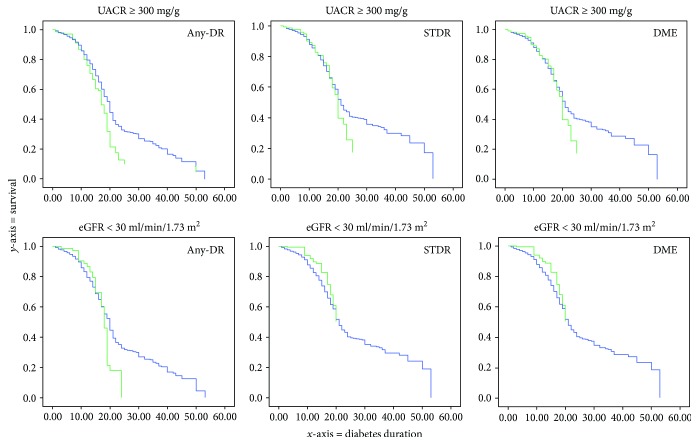
Plots of risk function of Cox study. The *y*-axis represents the accumulative risk to develop diabetic retinopathy, and the *x*-axis represents diabetes mellitus duration in years. The green curve represents the effect of patients with renal failure as a risk factor, and the blue curve patients without renal failure. At the top, there is the analysis of renal failure measured with UACR ≥ 300 mg/g, and at the bottom eGFR < 30 ml/min/1.73 m^2^.

In this paragraph, we compared survival study made with Kaplan-Meier product-limit graph and Cox proportional hazards model. In Figures 1 and 2, we show the accumulated risk of developing diabetic retinopathy in each of the three studied categories.

Figure 1 used the Kaplan-Meier product-limit graph to evaluate the relationship between renal failure diagnosed by UACR or eGFR in each DR type. Using the plots risk function, it is evident that UACR ≥ 300 mg/g increases significantly the accumulated risk of any-DR, STDR, and DME along with DM duration. The use of eGFR < 30 ml/min/1.73 m^2^ as a diagnosis of renal failure is not reliable as an indicator of an increased risk of STDR and DME, and a significant link appeared only if we studied all patients with any-DR.

Figure 2 shows the accumulated risk of DR using the Cox proportional hazards model. At the top of the figure, we observe an increased accumulated risk of development of any-DR, STDR, and DME in patients with renal failure using UACR ≥ 300 mg/g as method of diagnosis. On the contrary, below that, we observe that renal failure diagnosis using the eGFR < 30 ml/min/1.73 m^2^ was not significant for any of three types of DR. It is interesting that using the Kaplan-Meier curve, any-DR had an increased accumulative risk, but in the Cox's survival analysis, after the introduction of the other risk variables (age, sex, arterial hypertension, and HbA1c), the effect disappeared and became not significant.

## 4. Discussion

The present study should be taken in the context of previous studies [13, 17] conducted on the same population. Incidence of any-DR at the ten-year follow-up was 28.24% with a mean annual incidence of 8.21 ± 0.60% (7.06%–8.92%), similar to previously published data. Referable DR described as STDR had an annual incidence of 2.65 ± 0.14% (2.48%–2.88%), and DME had an annual incidence of 2.21 ± 0.18% (2%–2.49%); both results are similar to those of The Scottish National Diabetic Retinopathy Screening Programme [18]. A particularly interesting result from our study is that STDR incidence at ten years was 1451 patients (9.17%), and DME affected 1214 patients (7.67%), but only 237 patients had STDR without DME, representing 16.33% of patients with STDR. We should take into account that STDR might be due to DME or ischemic retina secondary to severe DR and conclude that only 1.50% of patients in our study developed STDR without DME. Due to this little difference in incidence between STDR and DME patients, the two groups have similar results according to risk factors and numerical statistical data.

For diabetic nephropathy (DN), with UACR ≥ 30 mg/g, there was 23.82% of patients who developed DN at the end of study, with a mean annual incidence of 7.02 ± 0.05% (6.97%–7.09%). On the contrary, with eGFR < 60 ml/min/1.73 m^2^, there was 20.06% patients with DN at the end of the study, with a mean annual incidence of 5.89 ± 0.12% (5.70%–6.13%). These discrepancies can be explained because glomerular filtration rate increases in initial diabetic renal failure and then reduces in most advanced nephropathy.

Statistical analysis shows that glomerular filtration was a significant risk factor for DR and can be a predictive factor for severe DR complications, as defined in our study as STDR and DME. However, introducing UACR as a risk factor in the equation in Cox's survival analysis, the eGFR became less significant; therefore, in the development of any-DR, the HR decreases from 1.854 to 1.223, with a change in significance from *p* = 0.002 to *p* = 0.04; Figures 1 and 2 clearly show this effect. When we use the Kaplan-Meier curve, the eGFR is a significant risk, but with the introduction of the other variables of age, sex, arterial hypertension, and HbA1c in the Cox's survival analysis, the effect of eGFR disappeared and UACR is the only effective risk factor. We can conclude, therefore, that UACR has greater relationship with DR development than eGFR does.

The study of STDR and DME also shows that UACR is a more significant risk factor than eGFR, despite eGFR being an important risk factor, with HR having similar values to other variables like arterial hypertension or insulin treatment.

We can explain that eGFR is a less significant risk factor than UACR in two ways. Firstly, changes in eGFR occur prior to an increase in UACR, increasing its filtration ratio in the early stage of diabetes mellitus and then reducing in advanced stages, reflecting the decline in renal function. Secondly, arteriosclerosis, which can be developed in parallel to diabetes, can decrease the glomerular filtration rate in patients with normoalbuminuria [19]. Both mechanisms can act as confounding factors in statistical results.

The greater significance of UACR in type 2 DM compared with type 1 DM (T1DM), which we encountered in a previous study with a series of T1DM patients [17], might be explained by the different methodologies used.

In another previously published study [20], we reported low significance of microalbuminuria as risk factor for DR in T2DM, but in the same study, we encountered significant values of albuminuria ≥ 300 mg/g and DR development. Again, this might be due to the different methodology and the fact that the previous study was a cross-sectional feature rather than a prospective ten-year follow-up as this present study is. In previous study, we defined microalbuminuria as 30–299 mg/g, and in the present study, we defined renal nephropathy if UACR was up 30 mg/g, which includes patients with microalbuminuria and macroalbuminuria.

Our results can be compared, though with caution, with similar published studies. Man et al. [21], in a case series study of 263 Caucasian patients, found a link between impaired renal function, measured by the CKD-EPI equation and severe forms of DR, but not with DME. However, as those authors said, perhaps its lower number of patients with DME can lower the significance of the results, whereas our study has a greater sample of patients (1214 patients with DME), which might highlight its significance.

Wu et al. [22] reported that levels of eGFR less than or equal to 99.4 ml/min/1.73 were significant for DR in a case-control hospital-based study. The present study considered only levels of eGFR less than 60 ml/min/1.73 as pathologic, because, as we have said, glomerular filtration increases in early stages of renal malfunction and can confound the results.

Most similar to our results are those reported by Rodríguez-Poncelas et al. [23], who carried out a cross-sectional study based on 28,344 patients. They demonstrated that prevalence of DR increases in patients with UACR ≥ 300 mg/g with and odds ratio of 2.0 and with a positive relation between the decrease in eGFR and STDR. The present study also demonstrates that eGFR is a less significant risk factor than UACR.

Other studies used the modification of diet in renal disease (MDRD-4 or MDRD-IDMS) [11] to determine eGFR. López et al. [24], in a cross-sectional study, used the MDRD-4 formula. Their results are similar to ours with a significance at *p* < 0.001, OD = 2.0, and 95%IC = 1.6–2.4 in a clinical series of 14,266 patients. Despite MDRD only being able to be used as an alternative to the CKD-EPI equation, some publications currently estimate that the latter provides a more accurate estimate of the eGFR [12, 25, 26].

The strengths of our study are the screening programme itself, which had included an 85.33% of T2DM patients in our HCA, and the ten-year follow-up of our T2DM population, yielding a large amount of data.

The limitations of our study are that we determined the CKD-EPI equation using creatinine data. Despite serum creatinine measurements being carried out in the same clinical laboratory, which used integrated database management system-traceable samples to minimize calibration bias, it can cause errors in determination. Also, patients with STDR, and/or DME, can be visited in hospital and bypass the screening programme, us affecting the statistical analysis.

## 5. Conclusions

In conclusion, the present study has shown that UACR have higher association than eGFR with diabetic retinopathy and its severe forms. From our data, we would encourage further studies to determine the glomerular filtration rate and the relationship of DR in T2DM patients as risk factor.

## Figures and Tables

**Table 1:** Descriptive and frequency values of the sample.

Year	Number of subjects screened (*n*)	Gender: men (%)	Mean age (years)	Diabetes duration (years)	Mean level of HbA_1c_ % mean ± SD (min–max)
2007	4910	2881 (57.31)	64.62 ± 12.23	8.37 ± 6.92	7.37 ± 1.48 (3.9–14)
2008	4873	2802 (56.16)	66.27 ± 12.32	8.66 ± 6.78	6.82 ± 1.24 (4.3–12)
2009	5191	2890 (54.41)	65.39 ± 12.41	8.57 ± 6.12	7.02 ± 1.7 (3.8–15)
2010	5243	3007 (56.03)	65.69 ± 11.7	8.23 ± 6.81	7.47 ± 1.5 (4.5–14.5)
2011	5264	2933 (55.60)	65.22 ± 12.12	8.29 ± 6.56	7.3 ± 1.5 (4–15.5)
2012	6193	3594 (56.72)	65.33 ± 12.08	8.23 ± 6.82	7.63 ± 1.4 (4.3–15.8)
2013	5494	3131 (55.69)	65.87 ± 12.07	8.28 ± 6.11	7.62 ± 1.41 (4.3–15.8)
2014	5983	3511 (57.33)	65.88 ± 11.94	8.34 ± 6.83	7.64 ± 1.4 (4–15.6)
2015	5026	2817 (56.05)	65.84 ± 12.39	8.35 ± 6.77	7.61 ± 1.5 (4.2–15)
2016	5423	3036 (56)	65.94 ± 12.27	8.32 ± 6.72	7.63 ± 1.4 (4.4–15.4)

Descriptive values are presented as number or mean ± standard deviation (SD).

**Table 2:** Incidence of diabetic retinopathy and renal diabetic disease.

Year	Any-DR, *n* (%)	STDR, *n* (%)	DME, *n* (%)	eGFR < 60 ml/min/1.73, *n* (%)	UACR ≥ 30 mg/g, *n* (%)	UACR ≥ 300 mg/g and eGFR< 30 ml/min/1.73 m^2^, *n* (%)
2007	390 (7.94)	131 (2.6)	104 (2.00)	294 (5.98)	343 (6.98)	3 (0.06)
2008	384 (7.88)	125 (2.5)	101 (2.02)	290 (5.95)	344 (7.06)	2 (0.04)
2009	411 (7.06)	132 (2.48)	112 (2.11)	301 (5.79)	363 (6.99)	3 (0.06)
2010	424 (8.05)	134 (2.49)	114 (2.12)	309 (5.89)	372 (7.09)	4 (0.07)
2011	407 (7.73)	141 (2.67)	110 (2.08)	310 (5.89)	368 (6.99)	4 (0.07)
2012	533 (8.6)	170 (2.68)	150 (2.36)	353 (5.70)	432 (6.97)	4 (0.06)
2013	489 (8.9)	162 (2.88)	135 (2.40)	324 (5.89)	390 (7.09)	4 (0.07)
2014	529 (8.84)	174 (2.84)	153 (2.49)	357 (6.13)	419 (7.01)	5 (0.08)
2015	415 (8.25)	139 (2.76)	122 (2.42)	301 (5.98)	351 (6.98)	4 (0.07)
2016	484 (8.92)	144 (2.65)	114 (2.10)	314 (5.79)	385 (7.09)	3 (0.05)

**Table 3:** Statistical analysis at the end of the ten-year follow-up study.

Variables	Category	Mean values	Univariate statistical study significance
Age (years)	No diabetic retinopathy	62.53 ± 13.63	*p* < 0.001, (*F* = 6.998)^∗^
	Diabetic retinopathy	71.94 ± 10.56	

Sex (male)	No diabetic retinopathy	41.50%	*p* < 0.001, OR 2.24 (1.05–3.22)^∗∗^
	Diabetic retinopathy	58.50%	

Diabetes duration (years)	No diabetic retinopathy	9.36 ± 6.58	*p* < 0.001, (*F* = 21.56)
	Diabetic retinopathy	12.18 ± 6.21	

Arterial hypertension	No diabetic retinopathy	49.53	*p* = 0.003, OR 2.04 (1.14–3.11)
	Diabetic retinopathy	50.47	

Insulin treatment	No diabetic retinopathy	18.54	*p* < 0.001, OR 3.98 (2.24–5.17)
	Diabetic retinopathy	51.40	

HbA1c (%)	No diabetic retinopathy	7.69 ± 2.69	*p* < 0.001, (*F* = 13.75)
	Diabetic retinopathy	8.07 ± 1.76	

eGFR (ml/min/1.73 m^2^)	No diabetic retinopathy	89.66 ± 16.11	*p* = 0.004, (*F* = 9.10)
	Diabetic retinopathy	56.32 ± 12.20	

UACR (mg/g)	No diabetic retinopathy	4.82 ± 43.00	*p* < 0.001, (*F* = 14.81)
	Diabetic retinopathy	75.30 ± 231.26	

^∗^Study made with two-tailed Student's *t*-test or ANOVA; *F*: Fisher-Snedecor distribution. ^∗∗^Study made with Chi-square; OR: odds ratio (95% CI).

**Table 4:** Multivariate analysis using Cox's proportional regression analysis of any-diabetic retinopathy.

Variables	Model 1^∗^	Model 2^∗∗^	Model 3^∗∗∗^	Model 4^∗∗∗∗^
Current age	*p* < 0.001	*p* < 0.001	*p* < 0.001	*p* < 0.001
	HR 1.136 (1.084–1.224)^†^	HR 1.140 (1.081–1.227)	HR 1.140 (1.072–1.284)	HR 1.145 (1.083–1.290)

Sex	*p* = 0.065	*p* = 0.077	*p* = 0.070	*p* = 0.073
	HR 0.761 (0.643–0.961)	HR 0.822 (0.668–1.060)	HR 0.820 (0.651–1.072)	HR 0.834 (0.644–1.015)

Insulin treatment	*p* < 0.001	*p* < 0.001	*p* < 0.001	*p* < 0.001
	HR 1.218 (1.131–1.448)	HR 1.302 (1.188–1.506)	HR 1.311 (1.174–1.501)	HR 1.302 (1.178–1.545)

Arterial hypertension	*p* = 0.05	*p* = 0.05	*p* = 0.045	*p* = 0.049
	HR 1.118 (0.985–1.788)	HR 1.121 (0.907–1.340)	HR 1.134 (1.002–1.161)	HR 1.367 (1.062–1.899)

HbA1c	*p* < 0.001	*P* < 0.001	*p* < 0.001	*p* < 0.001
	HR 2.135 (1.893–2.408)	HR 2.089 (1.811–2.632)	HR 2.052 (1.780–2.273)	HR 2.037 (1.806–2.297)

eGFR < 60 ml/min/1.73 m^2^		*p* = 0.002	*p* = 0.04	*p* = 0.09
		HR 1.854 (1.251–4.431)	HR 1.223 (1.098–1.201)	HR 1.103 (0.078–1.311)

UACR ≥ 30 mg/g			*p* < 0.001	*P* = 0.004
			HR 1.485 (1.103–1.548)	HR 1.485 (1.092–1.465)

UACR ≥ 300 mg/g and eGFR< 30 ml/min/1.73 m^2^				*p* < 0.001
				HR 1.998 (1.682–2.305)

Model 1^∗^, Cox's analysis in which the variables of renal function are not included; model 2^∗∗^, Cox's analysis in which we include only the eGFR variable for the study of renal function; model 3^∗∗∗^, Cox's analysis with eGFR and UACR as variables for the study of renal function; model 4^∗∗∗∗^, Cox's analysis in which we included in addition to the eGFR and the UACR, the presence of renal failure. ^†^Hazard ratio (95% CI).

**Table 5:** Descriptive data of diabetic retinopathy forms.

	No-DR	STDR	Significance	DME	Significance
Current age (years)	64.01 ± 13.99	64.05 ± 12.27	*p* = 0.005, *F* = 5.306	64.36 ± 12.84	*p* = 0.002, *F* = 6.401
DM duration (years)	8.71 ± 6.12	13.91 ± 7.85	*p* < 0.001, *F* = 26.141	13.77 ± 7.78	*p* < 0.001, *F* = 25.788
HbA1c (%)	7.33 ± 1.76	9.21 ± 1.97	*p* < 0.001, *F* = 29.033	9.18 ± 1.96	*p* < 0.001, *F* = 28.867
eGFR (<60 ml/min/1.73 m^2^)	85.69 ± 20.31	73.32 ± 24.09	*p* = 0.01, *F* = 8.494	73.17 ± 24.20	*p* = 0.01, *F* = 8.568
UACR (>30 mg/g)	3.25 ± 55.33	66.68 ± 172.56	*p* < 0.001, *F* = 14.640	67.06 ± 173.78	*p* < 0.001, *F* = 14.665

STDR: sight threatening diabetic retinopathy; DME: diabetic macular edema; *F* = Fisher-Snedecor distribution.

**Table 6:** Multivariate analysis using Cox's proportional regression analysis of STDR and DME.

	STDR	DME
Variables	Significance	Significance
	Hazard ratio (95% CI)^∗^	Hazard ratio (95% CI)

Current age	*p* = 0.018	*p* = 0.057
	HR 0.991 (0.984–0.999)	HR 0.993 (0.986–1.000)

Sex	*p* < 0.001	*p* < 0.001
	HR 0.644 (0.531–0.781)	HR 0.655 (0.539–0.794)

Insulin treatment	*p* = 0.01	*p* = 0.01
	HR 1.313 (1.123–1.535)	HR 1.308 (1.117–1.530)

Arterial hypertension	*p* < 0.001	*p* < 0.001
	HR 2.128 (1.726–2.623)	HR 2.126 (1.721–2.626)

HbA1c	*p* < 0.001	*p* < 0.001
	HR 3.230 (2.378–4.387)	HR 3.194 (2.350–4.340)

eGFR < 60 ml/min/1.73 m^2^	*p* = 0.07	*p* = 0.09
	HR 1.097 (0.899–1.979)	HR 1.033 (0.877–1.972)

UACR ≥ 30 mg/g	*p* = 0.004	*p* = 0.006
	HR 2.099 (1.163–3.072)	HR 1.978 (1.237–2.901)

UACR ≥ 300 mg/g and eGFR < 30 ml/min/1.73 m^2^	*p* < 0.001	*p* < 0.001
	HR 3.174 (2.140–4.708)	HR 3.190 (2.150–4.732)

^∗^Hazard ratio (95% confidence interval). STDR: sight threatening diabetic retinopathy; DME: diabetic macular edema. ^∗^Hazard ratio (95%CI).
